# Genetic Risk of MASLD in Mongolians: Role of *PNPLA3* and *FTO* SNPs

**DOI:** 10.3390/cimb47080605

**Published:** 2025-08-01

**Authors:** Yumchinsuren Tsedendorj, Dolgion Daramjav, Yesukhei Enkhbat, Ganchimeg Dondov, Gantogtokh Dashjamts, Enkhmend Khayankhyarvaa, Amin-Erdene Ganzorig, Bolor Ulziitsogt, Tegshjargal Badamjav, Batbold Batsaikhan, Shiirevnyamba Avirmed, Tulgaa Lonjid

**Affiliations:** 1 Department of Internal Medicine, Institute of Medical Sciences, Ministry of Economy and Development, Ulaanbaatar 16094, Mongolia; yumchinsuren.ims@mnums.edu.mn (Y.T.); dolgion.ims@mnums.edu.mn (D.D.); ganchimeg.ims@mnums.edu.mn (G.D.); gantogtokh.ims@mnums.edu.mn (G.D.); enkhmend.ims@mnums.edu.mn (E.K.); aminerdene.ims@mnums.edu.mn (A.-E.G.); bolor.ims@mnums.edu.mn (B.U.); tegshjargal.ims@mnums.edu.mn (T.B.); batbold.ims@mnums.edu.mn (B.B.); 2 Department of Health Research, Graduate School, Mongolian National University of Medical Sciences, Ulaanbaatar 14210, Mongolia; shiirevnyamba@mnums.edu.mn; 3 Department of Genetic Laboratory, Institute of Biology, Mongolian Academy of Sciences, Ministry of Economy and Development, Ulaanbaatar 16094, Mongolia; esubat2011@gmail.com

**Keywords:** fatty liver, genotype, MASLD, obesity

## Abstract

Background: This study aimed to determine the association between *PNPLA3* rs738409, rs2896019, and *FTO* rs9939609, rs17817449 single-nucleotide polymorphisms and the risk of metabolic dysfunction-associated steatotic liver disease (MASLD) in Mongolian individuals. Methods: We conducted a case-control study, enrolling 100 MASLD patients and 50 subjects without MASLD. We used the PCR-RFLP technique on three genotype SNPs (rs738409, rs2896019 in *PNPLA3*, and rs9939609 in *FTO*). We analyzed liver function and lipid metabolism parameters in the peripheral blood of study participants. A *p*-value below 0.05 was considered a statistically significant result. Results: This study, which included 150 participants aged 23 to 75, had a mean age of 46.73 ± 11.45 years, with 40% of participants being male (60 individuals). We observed the rs738409 (G), rs2896019 (G), and rs9939609 (A) alleles at a statistically significantly enhanced frequency in the case group (32.5%, 33%, and 21%) compared to the control group (19%, 25%, and 19%), indicating an increased risk of MASLD. The *FTO* rs17817449 SNP did not show a significant difference between groups. *PNPLA3* rs738409 GC/GG genotype (OR = 2.39, *p* = 0.019) and *FTO* rs9939609 AT/AA (OR = 2.55, *p* = 0.025) genotype showed a significant association with MASLD. In the evaluation of the *FTO* rs9939609, rs17817449, and *PNPLA3* rs738409, rs2896019 single-nucleotide polymorphisms among the research individuals, 18.7% had no SNPs, 15.3% had one SNP, 29.3% had two SNPs, 25.3% had three SNPs, and 11.3% had four SNPs. The risk of MASLD increased significantly for individuals having four SNPs (OR = 4.23, *p* = 0.007). Conclusions: We found that *PNPLA3* rs738409 GC/GG genotype and *FTO* rs9939609 AT/AA genotype are strongly associated with an increased risk of MASLD. Notably, individuals with a higher rate of SNP number, had a significantly higher risk of MASLD.

## 1. Introduction

In the early 1980s, Dr. Jurgen Ludwig observed that some people who did not consume alcohol had liver damage similar to alcohol-related liver disease. He named this condition non-alcoholic fatty liver disease (NAFLD), which has since become a significant field of research on hepatic health [1]. In June 2023, a Delphi consensus involving several societies, namely the American Association for the Study of Liver Diseases (AASLD), the European Association for the Study of the Liver (EASL), and Asociación Latinoamericana para el Estudio del Hígado (ALEH), redefined the terms and criteria for metabolic dysfunction-associated steatotic liver disease (MASLD). MASLD is currently defined as liver steatosis by imaging or biopsy in a patient with one or more cardiometabolic risk factors with no alcohol consumption. This shows a more comprehensive understanding of the condition’s connection to metabolism and associated health risks [2,3]. MASLD has emerged as a leading cause of chronic liver disease globally. It is characterized by the accumulation of fat in the liver and is closely associated with cardiometabolic risk factors such as obesity, diabetes, and cardiovascular disease.

The prevalence of MASLD has been increasing significantly, posing a major global health challenge due to the rise of metabolic dysfunction, obesity, and sedentary lifestyles worldwide [3,4,5,6]. Between 1990 and 2019, the prevalence of MASLD among adults increased from 17.6% to 23.4% accounting for 1.24 billion cases [7]. Furthermore, Younossi et al. (2023) reported that 38% of adults worldwide had been diagnosed with MASLD; that number is expected to increase to 55% by 2040 [8]. Despite recent research on MASLD in Mongolia, there is a lack of comprehensive national prevalence studies.

MASLD is a multifactorial disease, so genetics, metabolic dysfunction, and environmental factors play roles in the increase of MASLD. Moreover, previous studies demonstrated that genetic factors influence the risk of MASLD. According to genome-wide association studies (GWAS), some genes such as *PNPLA3*, *TM6SF2, MBOAT7, GCKR*, *FTO*, and *HSD17B13* play important roles in metabolic pathways, especially liver steatosis and disease progression [9,10]. Notably, the *PNPLA3* (adiponutrin/patatin-like phospholipase domain-containing protein 3) gene is the main genetic factor in the MASLD [11,12,13,14]. The *PNPLA3* gene is located on the long arm of chromosome 22 of humans and is mainly expressed in the liver. It has 481 amino acids. The mutation of codon 148 from isoleucine to methionine by the cytosine-to-guanine substitution is known as rs738409 single nucleotide polymorphism (SNP). This mutation causes increased fat accumulation in the liver, liver inflammation, fibrosis, cirrhosis, HCC, and MASLD [15]. This *PNPLA3* gene changes adiponutrin function and disrupts lipid metabolism in liver cells. Thus, triglycerides accumulate in liver cells, ultimately leading to liver steatosis. This process drives the development of MASLD and may progress to more severe conditions such as fibrosis and cirrhosis. According to GWAS studies, the *PNPLA3* gene polymorphisms rs738409 and rs2896019 are associated with hepatic steatosis, inflammation, fibrosis, cirrhosis, and even hepatic cancer [11,16,17]. Adiposity and obesity are associated with the pathogenesis of MASLD. In addition, MASLD occurs in 75% of overweight individuals and 90% of those who are severely obese. Overweight and obesity are major public health problems and the fifth-leading risk factor for mortality worldwide. Globally, 2.5 billion adults were overweight in 2022. Of these, 890 million were obese. In Mongolia, 30.9% of the population is classified as overweight, while 18.5% is considered obese [18]. While several studies have shown a strong association between fat mass and obesity-associated (*FTO*) polymorphisms and obesity risk, only a limited number have investigated the genetic differences of *FTO* between MASLD and non-MASLD individuals. The *FTO* gene is located on chromosome 16 and functions as an alpha-ketoglutarate-dependent dioxygenase in deoxyribonucleic acid (DNA) demethylation [19]. FTO is widely expressed throughout the body, involving the nervous system, adipose tissue, and liver. Experimental investigations indicate that FTO overexpression causes MASLD by increasing oxidative stress and accumulation of lipids in human hepatocytes [20]. The *FTO* variants rs9939609 and rs17817449 have been related to metabolic disease. Rs9939609 polymorphism has been reported to be associated with metabolic-related diseases such as obesity [21,22,23,24] and MASLD [25,26]. The rs9939609 SNP is formed by the conversion from thymine (T) to adenine (A).

Numerous research studies have examined the correlation between MASLD and polymorphisms in the *PNPLA3* and *FTO* genes. However, previous research has yielded contradictory findings across different populations. The prevalence of *PNPLA3* rs738409, rs2896019, and *FTO* rs9939609, rs17817449 SNPs and their relation to MASLD have been identified in numerous countries worldwide; however, there is a lack of evidence of this to have been conducted in Mongolia. Thus, we aimed to determine the association between *PNPLA3* rs738409, rs2896019, and *FTO* rs9939609, rs17817449 SNPs and the risk of MASLD in Mongolian individuals.

## 2. Materials and Methods

### 2.1. Study Design and Participants

In 2023–2024, we conducted a case-control study and enrolled 100 MASLD patients as well as 50 without MASLD at a referral hospital in Ulaanbaatar, Mongolia. The study case group inclusion criteria consisted of individuals diagnosed with fatty liver by ultrasound, exhibiting low alcohol consumption (AUDIT-C score of 0–7), and possessing one or more cardiometabolic risk factors (overweight or obesity, prediabetes and diabetes mellitus (DM), hypertriglyceridemia, decreased HDL, and hypertension). We selected a control group of age- and gender-matched, relatively healthy individuals without MASLD and with low alcohol consumption (AUDIT-C score of 0–7). Moreover, the exclusion criteria included individuals under 18 years of age, pregnant women, those infected with hepatitis B or C viruses, individuals with liver cirrhosis or cancer, alcohol consumers, and those who declined to participate in the research. The study was conducted by the Declaration of Helsinki and approved by the Ethics Review Committee of the Ministry of Health of Mongolia on 11 January 2023 (approval number: 01). Informed consent was obtained from all individuals involved in the study.

Anthropometric measurements: We measured anthropometric assessments, including weight, height, waist circumference (WC), and BMI according to WHO standards. The weight was measured on a calibrated weighing scale and recorded to the closest one decimal point (0.1 kg). Also, we used a stadiometer to measure the height and WC, recording the results to the nearest 0.1 cm. Abdominal obesity was defined as a waist circumference of >90 cm for men and >80 cm for women. Subjects with a BMI cutoff point of 18.5 to 24.9 kg/m^2^ were considered normal, whereas those with a BMI cutoff point of ≥25.0 to 29.9 kg/m^2^ were considered overweight, and those with a BMI cutoff point of ≥30 kg/m^2^ or higher were considered obese. Blood pressure was assessed using electronic sphygmomanometers.

Biochemical measures: After obtaining written informed consent, fasting peripheral venous blood samples (5–10 mL) were collected from participants using sterile, single-use EDTA-coated vacuum tubes in accordance with standard biosafety and infection prevention protocols. Biochemical analyses were conducted at the Central Research Laboratory of the Institute of Medical Sciences using the Mindray BS230 automated biochemical analyzer. The aspartate aminotransferase (AST), alanine aminotransferase (ALT), GGT (gamma-glutamyl transferase), fasting blood sugar (FBS), triglycerides (TG), total cholesterol (TC), serum low-density lipoprotein (LDL), and serum high-density lipoprotein (HDL) were measured for all participants.

### 2.2. Genotyping of PNPLA3 and FTO SNPs

**Figure 1:**
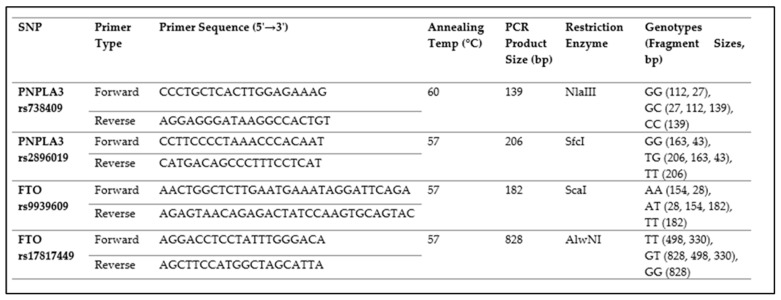
PCR-RFLP: primer sequence, enzyme, and genotypes.

The study obtained 1 mL of whole blood from each participant, which was kept at −20 °C until the DNA extraction process. DNA was isolated using the AccuPrep Genomic DNA extraction kit (Bioneer, Daejeon, Republic of Korea), according to the manufacturer’s instructions. The genotyping of four SNPs (rs738409, rs2896019 in *PNPLA3*, and rs9939609, rs17817449 in *FTO*) was conducted using the polymerase chain reaction–restriction fragment length polymorphism (PCR-RFLP) technique. For each SNP, PCR amplification was performed with specific primers. Electrophoresis of digested PCR products on a 3% agarose gel revealed distinct banding patterns that were used to determine the genotypes. The genetic constitution of *PNPLA3* rs738409, rs2896019, and *FTO* rs9939609, rs17817449 was determined using restriction enzyme length polymorphism with the rapid restriction enzymes Nla III, SfcI, ScaI, and AIwNI, respectively (Figure 1). All samples were independently analyzed in triplicate.

### 2.3. Statistical Analysis

All statistical analyses were performed using SPSS (version 23.0, Chicago, IL, USA). Normality was assessed both graphically (histograms) and with statistical tests (Kolmogorov–Smirnov test). For the analysis, descriptive statistics were computed for all variables of interest: mean ± standard deviations for variables with normal distributions, and median (IQR) for variables with non-normal distributions within each group. Categorical variables were described using numbers and proportions, and differences between groups were assessed using the chi-square (χ^2^) test or Fisher’s exact test, as appropriate. For comparing continuous variables between groups, the independent *t*-test and the non-parametric Mann–Whitney U test were used. Logistic regression analysis was performed to evaluate the risk factors and genetic factors of MASLD. Odds ratios (OR) with 95% confidence intervals (95% CI) were computed. A *p*-value less than 0.05 was considered to represent a statistically significant factor.

## 3. Results

In the study, 150 participants were aged from 23 to 75, with a mean age of 46.73 ± 11.45 years; 40% were male (60 individuals). The age group with the greatest participation was 40 to 49, accounting for 32% of participants. There were no significant differences observed by age group. (*p* = 0.994) (Table 1). Body mass index and waist circumference were significantly higher in the case group than in the control group (*p* < 0.001). Specifically, WC was 97.0 ± 18.83 cm in the case group and 73.60 ± 9.06 cm in the control group (*p* < 0.001). Additionally, mean systolic and diastolic blood pressure were statistically significantly higher in the case group compared to the control group (*p* = 0.034, *p* = 0.011). Regarding cardiometabolic risk factors for MASLD, the frequency of obesity, hypertriglyceridemia, decreased HDL, hypertension, prediabetes, and type 2 diabetes mellitus (DM) was statistically significantly different in the case group compared to the control group. Biochemical analysis showed statistically significant differences in the average levels of ALT, GGT, LDL, and triglycerides in the case group compared to the control group. Conversely, the average HDL levels were lower in the control group compared to the case group (Table 1).

### 3.1. PNPLA3 rs738409, rs2896019 and FTO rs9939609, rs17817449 Polymorphisms in the Study Individuals

**Figure 2:**
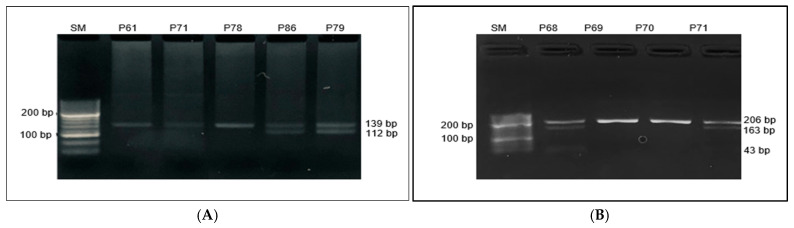
PCR-RFLP results of *PNPLA3* gene: (**A**) rs738409 C>G polymorphism (The size marker (SM) is shown on the left of the gel image, with the following genotypes identified: CC genotype (samples 61, 71, and 78) and CG genotype (samples 72, 86, and 79). The genotyping results are represented by distinct banding patterns for each genotype. (**B**) rs2896019 T>G polymorphism (The size marker (SM) is shown on the left of the gel image, with the following genotypes identified: TT genotype (samples 69 and 70) and TG genotype (samples 68 and 71). The genotyping results are represented by distinct banding patterns for each genotype.

Table 2 shows the frequencies of the *PNPLA3* rs738409, rs2896019, and *FTO* rs9939609, rs17817449 SNPs in the study subjects. *PNPLA3* rs738409: 44.7% had the CC genotype, 54.7% had the GC genotype, and 0.7% had the GG genotype in all study participants. The CC genotype was more common in the control group (62%) compared to the case group (36%) (*p* < 0.001). The CC genotype is associated with a reduced risk of MASLD (OR = 0.345, *p* < 0.001). Individuals with the GC/GG genotype had 68% higher MASLD, suggesting a functional association (OR = 2.9, *p* < 0.003), (see Table 2, Figure 2A)

Among the study participants, the genotypic frequency of *PNPLA3* rs2896019 was 36.7% TT, 60.7% were TG, and 2.7% were GG. When comparing the cases and controls, the TT genotype was more prevalent in the controls (48%) than in the cases (31%), with a statistically significant difference (*p* = 0.043). The TG/GG genotypes were present in 69% of the cases and 52% of the controls, showing a significant difference (*p* = 0.042, OR = 2.055, 95% CI: 1.022–4.130) (Table 2 and Figure 2B).

**Figure 3:**
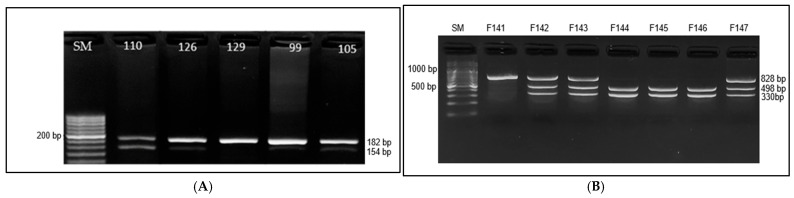
PCR-RFLP results of the *FTO* gene: (**A**) (rs9939609) T>A polymorphism. The size marker (SM) is shown on the left of the gel image, with the following genotypes identified: TT genotype (samples 129, 49) and AT genotype (samples 110, 126, 99, and 105). The genotyping results are represented by distinct banding patterns for each genotype. (**B**) (rs17817449) T>G polymorphism. The size marker (SM) is shown on the left of the gel image, with the following genotypes identified: TT genotype (samples 142, 143, and 147), TG genotype (samples 144, 145, and 146), and GG genotype (sample141). The genotyping results are represented by distinct banding patterns for each genotype.

The frequency of the *FTO* rs9939609 genotype and allele is shown in Table 3 and Figure 2. The genotype of heterozygosity for AT alleles in the case and control groups was 43% and 24%, respectively (*p* = 0.025). Additionally, the proportion of the AA allele polymorphism was 4% in the case group, while it was absent in the control group (Table 2, Figure 2A). Additionally, the *FTO* rs17817449 genotype was identified as TT in 62.7%, TG in 32%, and GG in 5.3% of the total individuals. The distribution of TT, TG, and GG genotypes did not differ significantly between groups (Table 2, Figure 3B).

We observed the rs738409 (G), rs2896019 (G), and rs9939609 (A) alleles at a statistically significant enhanced rate in the case group (32.5%, 33%, and 21%) compared to the control group (19%, 25%, and 19%), indicating an association with MASLD (Table 2).

### 3.2. Association of SNPs with the Anthropometric Parameters

We observed that the body measurements, specifically weight, BMI, and waist circumference, were significantly higher in individuals carrying the rs738409 GC/GG, rs2896019 TG/GG, and rs9939609 AT/AA genotypes. In contrast, there was no significant difference in systolic and diastolic blood pressure. In addition, individuals with the rs17817449 TG/GG genotype showed higher body weight and BMI compared to those with the TT genotype (*p* = 0.022, 0.039) (Table 3).

### 3.3. Correlation of SNPs with Biochemical Measures

Individuals with the *PNPLA3* rs738409 GC/GG and rs2896019 TG/GG genotypes showed significantly elevated levels of ALT and LDL compared to those with the rs738409 CC and rs2896019 TT genotypes, respectively, while the average HDL levels were lower in persons with the rs738409 GC/GG genotypes when compared to those with the CC genotype.

However, there were no significant differences in the mean levels of FSG, AST, TG, and TC (Table 3).

For the *FTO* rs9939609, the mean LDL value was statistically substantially elevated in persons with the AT/AA genotype relative to those with the TT genotype. Concurrently, average HDL levels were lower in persons with the AT/AA genotypes compared to those with the TT genotype (Table 3). Regarding the *FTO* rs17817449 genotype, the mean LDL level in individuals with the TG/GG genotype was 3.72 ± 0.94 mmol/L, which was significantly higher compared to individuals with the TT genotype (3.30 ± 0.74 mmol/L) (*p* = 0.003). Nevertheless, there were no statistically significant differences in the mean levels of glucose, triglycerides, HDL, and TC (Table 3). Furthermore, ALT, AST, and GGT levels showed no statistically significant differences between the groups when evaluating liver function (*p* = 0.789, *p* = 0.409, *p* = 0.365) (Table 3).

### 3.4. Comparison of Anthropometric and Laboratory Characteristics According to the Number of SNPs Detected

**Figure 4:**
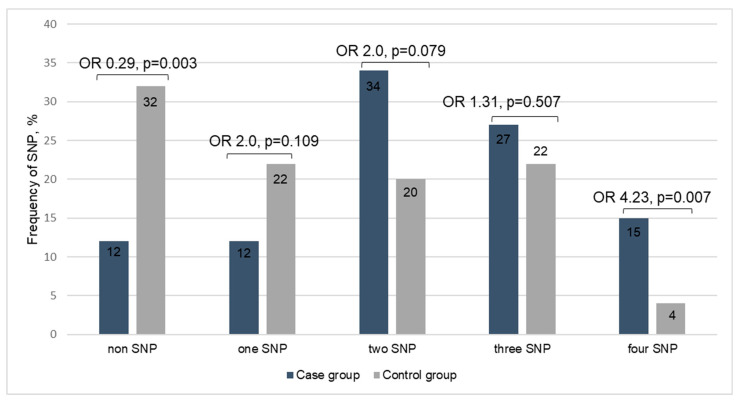
Frequency of SNP in *PNPLA3* rs738409 GC/GG, rs2896019 TG/GG and *FTO* rs9939609 AT/AA, rs17817449 TG/GG.

When evaluating the number of *FTO* rs9939609, rs17817449, and *PNPLA3* rs738409, rs2896019 SNPs detected in the study participants, the distribution was as follows: 18.7% had a non-SNP, 15.3% had one SNP, 29.3% had two SNPs, 25.3% had three SNPs, and 11.3% had four SNPs. The risk of MASLD increased in individuals having four SNPs (OR = 4.23, *p* = 0.007) (Figure 4). Regarding physical measurements, significant increase sin body weight, WC, and BMI were observed in individuals with a higher number of detected SNPs (*p* < 0.001). Additionally, differences in lipid metabolism markers, including LDL, HDL, and triglycerides, were also seen between the groups. The average HDL level was reduced as the number of discovered SNPs increased, in contrast to the group with lower detected SNPs (*p* = 0.001). Furthermore, liver function tests showed differences between groups in ALT and GGT (*p* = 0.029, 0.020) (Table 4).

### 3.5. MASLD-Related Risk Factors Using Univariate and Multivariate Logistic Regression Analysis

According to univariate logistic regression analysis (Table 5), several factors were significantly associated with MASLD (*p* < 0.005): arterial hypertension, prediabetes, or diabetes mellitus, decreased HDL, hypertriglyceridemia, the *PNPLA3* rs738409 GC/GG genotype, and the *PNPLA3* rs2896019 TG/GG genotype. In multivariate analysis, several factors demonstrated significant associations with MASLD. For Model 1, multivariate regression analysis identified arterial hypertension (OR = 2.59, 95p = 0.016), decreased HDL (OR = 3.12, *p* = 0.019), and the *PNPLA3* rs738409 GC/GG genotype (OR = 2.39, *p* = 0.019) as significant independent predictors of MASLD. Prediabetes or DM, hypertriglyceridemia, and the *PNPLA3* rs2896019 TG/GG genotype did not demonstrate statistical significance in this model. In Model 2, arterial hypertension (OR = 2.79, CI: 1.29–6.03, *p* = 0.009), decreased HDL (OR = 3.27, CI: 1.23–8.64, *p* = 0.017), and *FTO* rs9939609 AT/AA genotype showed a significant association with MASLD. Conversely, prediabetes or DM and hypertriglyceridemia were not important in this model.

## 4. Discussion

We analyzed the *PNPLA3* rs738409, rs2896019, and the *FTO* rs9939609, rs17817449 SNPs in two population groups: individuals with MASLD and a control group. To our knowledge, this is the first investigation of the correlation between MASLD and polymorphisms in the *PNPLA3* and *FTO* genes among Mongolian subjects. According to our study, the *PNPLA3* rs73809 G mutant allele was found in 32.5% of the case group compared to 19% of the control group, while the rs2896019 G mutant allele was present in 37% of the case group and 25% of the control group. These results are similar to several previous studies [27,28,29,30]. For example, Chen Li and colleagues (2019) reported a significantly higher frequency of the mutant G allele of *PNPLA3* I148M in the case group (54.6%) compared to the control group (35%) in the Qingdao Han ethnicity in China [27]. Similarly, Hotta et al. (2010) [31] discovered that 43% of MASLD patients in Japan had the rs738409 G allele, compared to 20% of the control group, showing a 23% difference between them [31], which is similar to the 13.5-point difference we found in our study. In a European study, Romeo et al. (2008) found the G allele in 39% of MASLD patients and 23% of the control group [32], while Kantartzis et al. (2009) identified it in 47% of German MASLD patients and 23% of controls [33]. Similarly, Kotronen et al. (2009) found the G allele in 40% of Finnish patients and 22% of healthy controls [30]. Compared to these studies, our results from the Mongolian population showed a similar pattern with more MASLD cases having the rs738409 G allele than the controls, but it was found less frequently in both groups overall. This finding reflects the unique genetic origins of this ethnic and population group and suggests that further genetic epidemiological studies are needed to determine whether these differences in Central Asian populations affect disease susceptibility and progression. Additionally, Elmansoury et al. (2024) found that the CG genotype increased the risk of liver disease by 1.6 times, and the GG genotype increased it by 3.2 times [34]. In our study, the relatively low occurrence of the GG genotype may be due to the small number of participants. To enhance the accuracy and reliability of future studies, we recommend increasing the number of participants in both the case and control groups. Research on the rs2896019 polymorphism is still limited. However, existing studies, including those involving both adult and pediatric populations, showed that the TG and GG genotypes are associated with an increased risk of MASLD [35,36]. Although GWAS proved the correlation between *PNPLA3* and MASLD, the prevalence and effect of this gene differ among populations and ethnicities [37,38]. A large meta-analysis found that the G allele of rs738409 was the most associated with MASLD [39]. Liu et al. and a meta-analysis by Sookoian et al. demonstrated that the rs738409 SNP of the *PNPLA3* gene increases the risk of liver fat accumulation, liver fibrosis, and cirrhosis due to MASLD [37,40]. In addition to rs738409, other SNPs, such as rs2281135, have been associated with NAFLD in African, Caucasian, East Asian, and Mexican Americans, while rs139051 is associated with an increased risk of NAFLD in the Han Chinese population [41,42]. Furthermore, *PNPLA3* rs2294918 has been demonstrated to be correlated with NASH in European and American cohorts. The *PNPLA3* gene variant rs2896019 is genetically linked to the well-researched rs738409 SNP, which is known to raise the risk of MASLD [43,44].

*FTO* gene rs9939609, rs1121980, rs17817449, rs8050136, rs9940128, rs8061518, rs9921255, and rs1477196 genotypes are associated with obesity, metabolic syndrome, and type 2 diabetes [44,45,46,47].

While several studies have shown a strong association between *FTO* polymorphisms and obesity risk [48], only a limited number have investigated the genetic differences of *FTO* between MASLD. According to Zhan Gu et al., allele A of rs9939609 was associated with increased MASLD risk in the elderly [48,49,50]. Fawwad et al. observed an increase in central obesity associated with the A allele in Karachi, Pakistan [22]. According to Shabana et al., the frequency of the rs9939609 A allele was 29.3% in the obese group and 23.5% in the control group [51]. This is similar to the results of our investigation. For instance, in our study, the A allele of the rs9939609 SNP was present in 25.5% of the case group and 12% of the control group, indicating a statistically significant elevation in the case group (*p* = 0.008, OR = 2.51, 95% CI: 1.26–4.96). This finding may be associated with the modification of the participants’ BMI because our research case group consisted of individuals with a BMI over 25 kg/m^2^. Chen et al. conducted a study to investigate the correlation between *FTO* gene polymorphisms and the risk of MASLD. The study identified the *FTO* gene as associated with the risk of MASLD in obese males. However, there is no significant association between *FTO* gene polymorphisms and the risk of MASLD with a BMI of less than 25 kg/m^2^ [52]. As a predictor of metabolic disorders, the *FTO* gene plays a conclusive role in the control of energy balance and is highly expressed in many tissues, including fat and liver [53]. In our study, although several factors were significant predictors of MASLD in univariate regression analysis, arterial hypertension and decreased HDL were major predictors of MASLD in multivariate regression analysis. Moreover, genetic factors such as *PNPLA3* rs738409 GC/GG genotype and *FTO* rs9939609 AT/AA genotype were significantly associated with MASLD. There are several hypotheses that suggest that the Rs17817449 genotype is not associated with the population we studied. These include the following: - other strong genetic or environmental influences may mask the modest effect of this variant on MASLD risk; - the low statistical power due to the small sample size may have limited the ability to detect a robust association. The limitations of our research include the absence of a biopsy in the subjects, while the control group was relatively small. Our study focused on the *PNPLA3* and *FTO* gene polymorphisms, which included biochemical tests for liver function and lipid metabolism. However, we had the advantage of simultaneously identifying four SNPs in the *PNPLA3* and *FTO* genes in our study participants.

A research study in Italy, including people with the G allele of the rs738409 gene, revealed an association only with ALT levels, but in the Finnish population, only mean AST levels exhibited an association [30,31]. In contrast, serum AST/ALT levels in African Americans were not substantially correlated with the G gene [54]. According to our research, the presence of risk genotypes, such as rs738409 CG/GG and rs2896019 TG/GG, was associated with higher levels of ALT. The discrepancies in these results are likely due to variations in the individuals recruited throughout the various investigations, including adults with MASLD.

## 5. Conclusions

We found a strong link between *PNPLA3* rs738409 GC/GG and *FTO* rs9939609 AT/AA genotypes and increased risk of MASLD. Individuals with multiple risk variants showed higher body weight, waist circumference, BMI, as well as adverse lipid profiles. These genetic markers may contribute to steatotic liver development and lipid disorders. Genetic screening for these polymorphisms could help early identification of high-risk individuals, enabling targeted prevention and management to reduce MASLD impact.

## Figures and Tables

**Table 1:** Demographic and clinical characteristics of the study participants.

Characteristics	Total *n* = 150	Case *n* = 100	Control *n* = 50	*p* Value
Gender (female), *n* (%)	90 (60)	60 (60)	30 (60)	1
Age (years) *	46.73 ± 11.45	46.80 ± 11.60	46.73 ± 11.25	0.912
BMI (kg/m^2^) *	27.37 ± 5.51	29.83 ± 5.05	22.45 ± 1.94	<0.001
WC (cm) *	89.26 ± 19.62	97.00 ± 18.83	73.60 ± 9.06	<0.001
SABP (mmHg) *	124.51 ± 18.72	126.84 ± 19.23	119.79 ± 16.86	0.034
DABP (mmHg) *	82.70 ± 12.49	84.55 ± 12.36	78.87 ± 11.98	0.011
FSG (mmol/L) *	5.45 ± 1.96	5.75 ± 2.30	4.85 ± 0.74	0.008
ALT (IU/mL) **	21.68 (20.68)	23.25 (25.63)	17.49 (15.55)	0.005
AST (IU/mL) **	21.15 (12.49)	21.55 (16.12)	19.65 (8.44)	0.066
GGT	27.6 (37.9)	33.0 (39.2)	20 (14.7)	0.001
TG (mmol/L) *	1.55 ± 1.02	1.60 ± 1.01	1.45 ± 0.74	0.389
TC (mmol/L) *	5.59 ± 1.60	5.83 ± 1.76	5.11 ± 1.06	0.010
LDL (mmol/L) *	3.46 ± 0.84	3.59 ± 0.88	3.20 ± 0.69	0.008
HDL (mmol/L) *	1.48 ± 0.34	1.43 ± 0.33	1.58 ± 0.34	0.016
Smoking, *n* (%)	29 (19.3)	19 (19)	10 (20)	1
Obesity, *n* (%)	96 (64)	92 (92)	4 (8)	<0.001
Hypertriglyceridemia, *n* (%)	69 (46)	53 (53)	16 (32)	0.011
Decreased HDL, *n* (%)	57 (38)	48 (48)	9 (18)	<0.001
Hypertension, *n* (%)	87 (58)	68 (68)	19 (38)	<0.001
Prediabetes or DM, *n* (%)	46 (30.7)	37 (37)	9 (18)	0.013

* mean ± SD; ** median (IQR); BMI: body mass index; WC: waist circumference; SABP: systolic arterial blood pressure; DABP: diastolic arterial blood pressure; FSG: fasting serum glucose; ALT: alanine aminotransferase; AST: aspartate aminotransferase; GGT: gamma-glutamyl transferase; TG: triglycerides; TC: total cholesterol; LDL: low-density lipoprotein; HDL: high-density lipoproteins; DM: diabetes mellitus.

**Table 2:** Allele and genotype frequency of the rs738409, rs2896019 of *PNPLA3*, and rs9939609, rs17817449 of *FTO* in the study individuals.

Gene	Total *n* = 150 (%)	Case *n* = 100 (%)	Control *n* = 50 (%)	χ^2^	*p* Value	OR (95% CI)	*p* Value
*PNPLA3* rs738409, genotype frequency							
CC	67 (44.7)	36 (36)	31 (62)	9.117	0.003	0.345 (0.171–0.696)	0.003
CG	82 (54.7)	63 (63)	19 (38)	8.407	0.004	2.778 (1.379–5.598)	0.004
CG/GG	83 (55.3)	64 (64)	19 (38)	9.117	0.003	2.901 (1.437–5.853)	0.003
C allele	216 (72)	135 (67.5)	81(81)	6.027	0.014	0.487 (0.273–0.871)	0.015
G allele	84 (28)	65 (32.5)	19 (19)			2.053 (1.149–3.668)	0.015
*PNPLA3* rs2896019, genotype frequency							
TT	55 (36.7)	31 (31)	24 (48)	4.148	0.042	0.487 (0.242–0.978)	0.043
TG	91 (60.7)	65 (65)	26 (52)	2.361	0.124	1.714 (0.859–3.419)	0.126
TG/GG	95 (63.3)	69 (69)	26 (52)	4.148	0.042	2.055 (1.022–4.130)	0.043
T allele	201 (67)	126 (63)	75(75)	4.342	0.037	0.568 (0.332–0.970)	0.038
G allele	99 (33)	74 (37)	25 (25)			1.738 (1.016–2.973)	0.043
*FTO* rs9939609, genotype frequency							
TT	91 (60.7)	(53)	38 (76)	7.390	0.007	0.356 (0.167–0.760)	0.008
AT	55 (36.7)	43 (43)	12 (24)	5.182	0.023	2.389 (1.117–5.109)	0.025
AT/AA	59 (39.3)	47 (47)	12 (24)	7.390	0.008	2.808 (1.315–5.996)	0.008
T allele	237 (79)	149 (74.5)	88 (88)	7.324	0.007	0.398 (0.201–0.788)	0.008
A allele	63 (21)	51 (25.5)	12 (12)			2.510 (1.269–4.964)	
*FTO* rs17817449, genotype frequency							
TT	94 (62.7)	59 (59)	35 (70)	1.724	0.189	0.918 (0.455–1.852)	0.104
TG	48 (32)	33 (33)	15 (30)	0.138	0.710	1.090 (0.540–2.199)	0.086
TG/GG	56 (37.3)	41 (41)	15 (30)	1.724	0.189	1.090 (0.540–2.199)	0.104
T allele	237 (79)	157 (78.5)	80 (80)	0.090	0.764	0.913 (0.504–1.655)	0.115
G allele	63 (21)	43 (21.5)	20 (20)			1.096 (0.604–1.986)	0.115

**Table 3:** Associations of clinical characteristics and genotypes of *PNPLA3* rs738409, rs2896019 and *FTO* rs9939609, rs17817449.

Characteristics	*FTO* Gene						*PNPLA3* Gene					
	rs9939609			rs17817449			rs738409			rs2896019		
	TT *n* = 91	AT/AA *n* = 59	*p* Value	TT *n* = 55	TG/GG *n* = 95	*p* Value	CC *n* = 67	CG/GG *n* = 83	*p* Value	TT *n* = 55	TG/GG *n* = 95	*p* Value
Weight (kg) *	70.17 ± 15.17	81.90 ± 18.40	<0.001	72.28 ± 15.95	78.98 ± 19.11	0.022	68.73 ± 13.68	79.67 ± 18.65	<0.001	69.38 ± 13.99	77.92 ± 18.51	0.004
BMI (kg/m^2^) *	25.88 ± 4.59	29.68 ± 6.03	<0.001	26.65 ± 5.17	28.57 ± 5.88	0.039	25.58 ± 4.24	28.82 ± 5.99	<0.001	25.83 ± 4.61	28.26 ± 5.81	0.009
WC (cm) *	85.04 ± 18.37	96.48 ± 19.78	0.001	88.05 ± 18.74	91.48 ± 21.18	0.343	84.16 ± 17.57	93.38 ± 20.33	0.007	84.76 ± 17.39	91.73 ± 20.43	0.053
SABP (mmHg) *	122.85 ± 20.05	127.20 ± 16.12	0.180	124.16 ± 19.80	125.14 ± 16.72	0.768	122.14 ± 15.49	126.39 ± 20.85	0.180	122.14 ± 15.49	126.39 ± 20.85	0.180
DABP (mmHg) *	81.69 ± 12.47	84.31 ± 12.45	0.226	82.72 ± 12.99	82.65 ± 11.62	0.977	80.71 ± 12.57	84.25 ± 12.28	0.095	80.71 ± 12.57	84.25 ± 12.28	0.095
FSG (mmol/L) *	5.61 ± 2.44	5.21 ± 0.78	0.230	5.43 ± 2.02	5.49 ± 1.88	0.854	5.36 ± 1.99	5.52 ± 1.96	0.636	5.36 ± 1.99	5.52 ± 1.96	0.636
ALT (IU/mL) **	20 (17.8)	23.9 (28.1)	0.023	20.7 (19.7)	23.2 (27.8)	0.789	18.9 (14.5)	23.9 (28.3)	0.005	20 (14.3)	23.48 (29.2)	0.022
AST (IU/mL) **	20.78 (10.2)	21.4 (19)	0.151	20.7 (10.4)	21.5 (18.3)	0.409	20.2 (7.4)	22.14 (18.18)	0.022	20.78 (8.48)	21.80 (16)	0.078
GGT (IU/mL) **	24.5 (9.8, 247.5)	32.2 (11.9, 412)	0.011	27.1 (30.1)	29.6 (44.7)	0.365	21.6 (33.6)	31.2 (38.6)	0.028	23.6 (18.6)	33 (42.7)	0.004
TG (mmol/L) *	1.54 ± 1.13	1.57 ± 0.84	0.882	1.50 ± 1.06	1.63 ± 0.96	0.481	1.68 ± 1.21	1.45 ± 0.83	0.180	1.50 ± 0.91	1.58 ± 1.08	0.671
TC (mmol/L) *	5.54 ± 1.78	5.67 ± 1.26	0.616	5.59 ± 1.77	5.58 ± 1.16	0.971	5.56 ± 2.01	5.62 ± 1.18	0.819	5.37 ± 1.81	5.72 ± 1.46	0.199
LDL (mmol/L) *	3.33 ± 0.86	3.66 ± 0.77	0.018	3.30 ± 0.74	3.72 ± 0.94	0.003	3.29 ± 0.76	3.59 ± 0.88	0.030	3.23 ± 0.83	3.59 ± 0.82	0.012
HDL (mmol/L) *	1.53 ± 0.35	1.41 ± 0.30	0.032	1.50 ± 0.36	1.45 ± 0.30	0.312	1.56 ± 0.33	1.42 ± 0.33	0.008	1.47 ± 0.35	1.43 ± 0.32	0.616

* mean ± SD; ** median (IQR); BMI: body mass index; WC: waist circumference; SABP: systolic arterial blood pressure; DABP: diastolic arterial blood pressure; FSG: fasting serum glucose; ALT: alanine aminotransferase; AST: aspartate aminotransferase; GGT: gamma-glutamyl transferase; TG: triglycerides; TC: total cholesterol; LDL: low-density lipoprotein; HDL: high-density lipoproteins.

**Table 4:** Comparison of anthropometric and laboratory characteristics according to the number of SNPs detected.

Characteristics	Total *n* = 150	Non-SNP *n* = 28	One SNP *n* = 23	Two SNP *n* = 44	Three SNP *n* = 38	Four SNP *n* = 17	*p* Value
Weight (kg) *	74.7 ± 17.4	66.0 ± 12.5	68.1 ± 12.9	74.5 ± 16.3	77.3 ± 17.3	92.9 ± 19.1	<0.001
BMI (kg/m^2^) *	27.3 ± 5.51	24.5 ± 4.0	25.7 ± 3.9	27.0 ± 5.0	28.6 ± 5.0	32.1 ± 6.68	<0.001
WC (cm) *	89.2 ± 19.6	84.3 ± 18.2	79.3 ± 16.0	90.9 ± 18.7	108.0 ± 22.0	89.2 ± 19.6	<0.001
SABP (mmHg) *	124.5 ± 18.7	121.8 ± 15.1	124.9 ± 14.9	120.2 ± 14.9	128.1 ± 12.8	132.0 ± 20.3	0.171
DABP (mmHg) *	78.3 ± 8.9	84.2 ± 16.4	81.6 ± 12.2	81.6 ± 12.2	84.4 ± 10.7	86.5 ± 14.7	0.212
FSG (mmol/L) *	5.4 ± 1.9	5.7 ± 2.9	5.1 ± 1.0	5.4 ± 2.5	5.3 ± 0.9	5.4 ± 0.9	0.910
ALT (IU/mL) **	21.6 (20.6)	20.2 (13.8)	22.1 (13.3)	22.4 (29.3)	34.0 (20.2)	22.1 (40.0)	0.029
AST (IU/mL) **	21.1 (12.4)	19.3 (8.9)	20.7 (5.3)	21.2 (15.8)	20.8 (12.3)	31.0 (37.8)	0.067
GGT (IU/mL) **	27.6 (37.9)	19.4 (18.8)	28.4 (35.9)	25.8 (21.4)	33.6 (44.1)	35.7 (68.4)	0.020
TG (mmol/L) *	1.5 ± 1.0	1.5 ± 1.0	2.0 ± 1.5	1.2 ± 0.5	1.5 ± 0.8	1.8 ± 1.1	0.023
TC (mmol/L) *	5.5 ± 1.6	5.3 ± 2.2	5.9 ± 2.3	5.3 ± 0.8	5.6 ± 1.4	5.8 ± 1.1	0.559
LDL (mmol/L) *	3.4 ± 0.8	2.9 ± 0.6	3.5 ± 0.7	3.4 ± 0.8	3.5 ± 0.9	4.0 ± 0.6	0.001
HDL (mmol/L) *	1.4 ± 0.3	1.6 ± 0.3	1.5 ± 0.3	1.4 ± 0.3	1.4 ± 0.2	1.3 ± 0.2	0.019

* mean ± SD; ** median (IQR); BMI: body mass index; WC: waist circumference; SABP: systolic arterial blood pressure; DABP: diastolic arterial blood pressure; FSG: fasting serum glucose; ALT: alanine aminotransferase; AST: aspartate aminotransferase; GGT: gamma-glutamyl transferase; TG: triglycerides; TC: total cholesterol; LDL: low-density lipoprotein; HDL: high-density lipoproteins.

**Table 5:** MASLD-related factors using univariate and multivariate logistic regression analysis.

Characteristics	Univariate Analysis			Multivariate Analysis					
				Model 1 *			Model 2 **		
	OR	95% CI	*p* Value	OR	95% CI	*p* Value	OR	95% CI	*p* Value
Arterial hypertension	3.46	1.70–7.04	0.001	2.59	1.19–5.64	0.016	2.79	1.29–6.03	0.009
Prediabetes or DM	2.67	1.16–6.12	0.020	1.83	0.73–4.64	0.194	1.94	0.78–4.85	0.153
Decreased HDL	4.20	1.85–9.56	0.001	3.12	1.20–8.10	0.019	3.27	1.23–8.64	0.017
Hypertriglyceridemia	2.39	1.17–4.88	0.016	1.47	0.60–3.58	0.391	1.32	0.54–3.21	0.538
*PNPLA3* rs738409 (GC/GG)	2.90	1.43–5.85	0.003	2.39	1.02–5.62	0.045	-	-	-
*PNPLA3* rs2896019 (TG/GG)	2.05	1.02–4.13	0.043	1.23	0.52–2.94	0.629	-	-	-
FTO rs9939609 (AT/AA)	2.80	1.31–5.99	0.008	-	-	-	2.55	1.12–5.79	0.025

* Model 1: included the *PNPLA3* genetic variant; ** Model 2: included the *FTO* genetic variant; DM: diabetes mellitus; HDL: high-density lipoproteins.

## Data Availability

Data are contained within the article.
